# Dataset on an extended technology acceptance model: A combined application of PLS-SEM and NCA

**DOI:** 10.1016/j.dib.2023.109190

**Published:** 2023-04-29

**Authors:** Nicole Franziska Richter, Sven Hauff, Aleksandar Evgeniev Kolev, Sandra Schubring

**Affiliations:** a University of Southern Denmark, Denmark; b Helmut Schmidt University, Hamburg, Germany; c Hamburg University of Technology, Hamburg, Germany

**Keywords:** French consumer data, E-book reader, Consumer behavior, Technology acceptance model (TAM), Partial least squares structural equation modeling (PLS-SEM), Necessary condition analysis (NCA)

## Abstract

As technology has become indispensable in consumers’ daily life and economic growth, understanding how and why consumers decide to accept and use a new technology has become essential to both academic researchers and practice. This article provides a detailed dataset based on a questionnaire that utilizes an extended technology acceptance model (TAM), incorporating the theory of consumer values and the innovation diffusion theory. Data collection was done with an online survey among French consumers, resulting in a sample size of 174. The dataset contains measures on various consumer attitudes and perceptions (e.g., consumption values) that influence intention and behaviors (adoption intention and technology use). This article supplements a published research article by Richter, Schubring, Hauff, Ringle and Sarstedt [1] which provides a detailed guide on how to combine partial least squares structural equation modeling (PLS-SEM) with necessary condition analysis (NCA) and a related illustration in a standard software published by Richter, Hauff, Ringle, Sarstedt, Kolev and Schubring [2].

**Specifications Table**

**Table:** 

Subject	Management of technology and innovation, business research
Specific subject area	Consumer behavior, innovation diffusion, marketing, technology adoption
Type of data	Tables, figures, raw dataset
How the data were acquired	The data stems from a survey about the adoption intention and usage intensity of e-book readers. The survey included multi-item scales of different reflectively measured constructs that were adapted from prior research on technology use. The survey was distributed among French consumers with a quota sampling technique based on age, gender, education, income, and regional distribution criteria, which aimed at procuring a representative sample of France's general population. The data collection in France commenced on the 4th of March 2014 until the 12th of March 2014 and was carried out by Harris Interactive, an international market research agency.
Data format	Raw
Data source location	Data collection in France. Data collecting institution: Hamburg University of Technology, Institute of Human Resource Management and Organizations, Am Schwarzenberg-Campus 4, 21073 Hamburg, Germany in collaboration with Harris Interactive AG, Großneumarkt 50, 20459 Hamburg, Germany.
Data accessibility	The data is available via Mendeley. Schubring, Sandra; Richter, Nicole (2023), “Extended TAM”, Mendeley Data, V4, doi: 10.17632/pd5dp3phx2.4 It can be accessed via this link: https://data.mendeley.com/datasets/pd5dp3phx2
Related research article	Richter, N. F., Schubring, S., Hauff, S., Ringle, C. M., & Sarstedt, M. (2020). When predictors of outcomes are necessary: Guidelines for the combined use of PLS-SEM and NCA. Industrial Management + Data Systems, 120(12), 2243-2267. DOI:10.1108/IMDS-11-2019-0638

## Value of the Data

• This data contains information on key determinants of consumers’ intention to adopt and use a new technology. The analysis of the data can help to improve the general understanding of consumer behavior with respect to deciding whether to embrace and use innovative products.
• This data provides researchers with the core endogenous and exogenous constructs that capture technology adoption and usage in the context of e-book readers. Using the data, researchers can estimate revised versions of the models proposed in this article, run meta-analyses, and cross-cultural comparisons with additional data collected by them.
• This data provides insights for business practitioners involved in the technology industry who need to implement optimal diffusion strategies for their innovative market offerings.
• The data enables researchers interested in broadening their knowledge about the potential of combined applications of PLS-SEM and NCA to replicate in detail the step-by-step guidelines provided in Richter, N. F., Schubring, S., Hauff, S., Ringle, C. M., & Sarstedt, M. (2020). When predictors of outcomes are necessary: Guidelines for the combined use of PLS-SEM and NCA. Industrial Management + Data Systems, 120(12), 2243-2267, and Richter, N. F., Hauff, S., Ringle, C. M., Sarstedt, M., Kolev, A. E., & Schubring, S. (2023). How to apply necessary condition analysis in PLS-SEM. In H. Latan, J. F. Hair, & R. Noonan (Eds.), Partial Least Squares Path Modeling: Basic Concepts, Methodological Issues and Applications: Springer, which use this data to showcase the methods’ joint application.

## 1 Objective

**Fig. 1:**
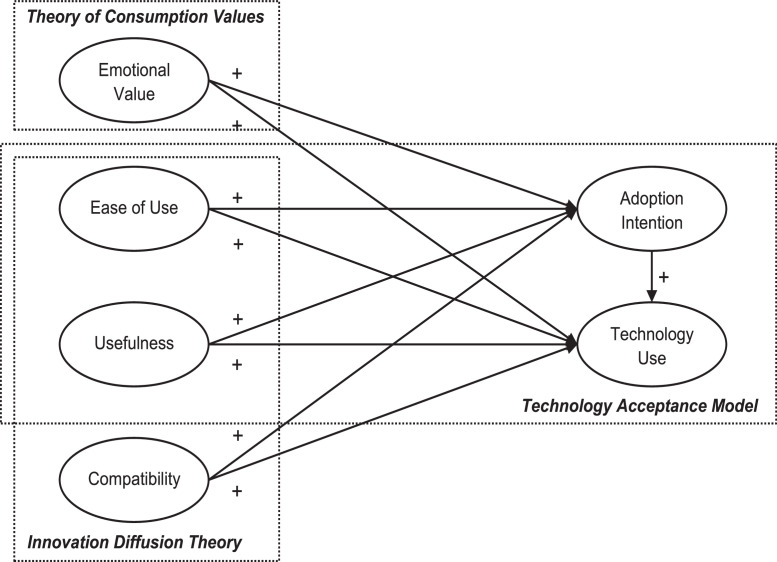
Conceptual model. *Source:* Adapted from Richter, Schubring, Hauff, Ringle and Sarstedt [1].

The data provided in this article represents a sample of 174 French consumers who were asked about their adoption intention and usage intensity of e-book readers. It was applied in a guideline and an illustration [1,2] that showcase the joint application of PLS-SEM [3,4] and NCA [5,6] using the model illustrated in Fig. 1. NCA is increasingly recognized in different fields [7,8]. NCA allows the identification of necessary conditions in data sets. A necessary condition is a constraint, a bottleneck, or a critical factor that must be overcome or satisfied for a desired outcome to exist. This corresponds to the logic of necessity according to which an outcome (or a certain level of an outcome) can only be achieved if the necessary cause is present (or at a certain level). Therewith, NCA can complement PLS-SEM analyses in which the interpretation of relationships between the determinants and the outcome often follows an additive sufficiency logic where multiple determinants are sufficient but not necessary to change the outcome (and can compensate for each other). The combined use of PLS-SEM and NCA is seen as a very promising avenue for future research [9] and is demonstrated in first applications [10]. The combined use of PLS-SEM and NCA enables researchers to explore and validate hypotheses following a sufficiency logic, as well as hypotheses drawing on a necessity logic. Using the data of this article, researchers can replicate the procedural steps discussed in Richter, Schubring, Hauff, Ringle and Sarstedt [1] and illustrated in Richter, Hauff, Ringle, Sarstedt, Kolev and Schubring [2] on their own.

## 2 Data Description

The datafile contains 19 columns (items) and 174 rows (observations). Items are named in accordance with their constructs as either abbreviations or acronyms of them. For example, ease of use (EOU) is measured by EOU_01, EOU_02, and EOU_03 (see Table 1). All items were measured with five-point Likert scales with higher values denoting higher levels of agreement—from one (“strongly disagree”) to five (“agree fully”). As an exception, technology use was measured with a single item (USE_01), which captures the respondent's frequency of reading e-books on a scale with the following range: one (“never”); two (“seldom”); three (“several times a month”); four (“once a week”); five (“several times a week”); six (“daily”); seven (“several times daily”). Table 1 provides the measurement item codebook and Table 2 provides the items’ descriptive statistics.

**Table 1:** Measurement item codebook.

Construct	Item	Indicator	Description	Source
Perceived Usefulness (reflective)	PU_1	General advantage	My e-book reader offers an advantage over conventional books.	[11,12]
	PU_2	Practical application	My e-book reader is more practical.	
	PU_3	Improvement of reading	Using an e-book reader improves the quality of my reading (e.g., font size).	
Compatibility (reflective)	CO_1	Reading behavior	My e-book reader is compatible with all aspects of my reading behavior (e.g., using the technology, touching, turning pages).	[12,13]
	CO_2	Consumption pattern	My e-book reader fits well with my consumer style (e.g., online book downloading).	
	CO_3	Reading needs	My e-book reader suits my reading needs (place, time, etc.; e.g., on holidays, on public transport, at home)	
Emotional Value (reflective)	EMV_1	Enjoyment	My e-book reader is one I enjoy.	[14]
	EMV_2	Pleasure	My e-book reader gives me pleasure.	
	EMV_3	Relaxation	My e-book reader helps relax me.	
Ease of Use (reflective)	EOU_1	Learning duration	I quickly learned how to operate my e-book reader.	[12]
	EOU_2	Operation	It is simple to operate an e-book reader.	
	EOU_3	Menu navigation	My e-book reader has a simple and clear menu navigation	
Adoption Intention (reflective)	AD_1	Future usage	I intend to continue using my e-book reader in the future.	[15]
	AD_2	Daily usage	I will always try to use my e-book reader in my daily life.	
	AD_3	Frequent usage	I plan to continue to use my e-book reader frequently.	
Technology Use (single item)	USE_1	Use	How frequently do you utilize the following electronic media with the aid of your e-book reader? e-books	[15]

*Note:* The question formulation for respondents who do not own an e-book reader and use alternative mobile electronic devices to read e-books substitutes “…e-book reader…” with “…mobile electronic device…”.

**Table 2:** Descriptive statistics.

Latent variable	Item	Mean	Range [Min; Max]	Standard deviation	Excess kurtosis	Skewness
Emotional value	EMV_01	3.902	[1; 5]	0.842	1.942	-1.036
	EMV_02	3.724	[1; 5]	0.887	0.940	-0.675
	EMV_03	3.799	[1; 5]	0.877	1.465	-0.933
Ease of use	EOU_01	4.011	[1; 5]	0.988	0.800	-0.996
	EOU_02	4.092	[1; 5]	0.811	0.798	-0.822
	EOU_03	3.971	[1; 5]	0.867	1.201	-0.904
Perceived usefulness	PU_01	3.753	[1; 5]	0.923	0.566	-0.768
	PU_02	3.397	[1; 5]	0.970	-0.176	-0.296
	PU_03	3.598	[1; 5]	1.055	-0.106	-0.585
Compatibility	CO_01	3.299	[1; 5]	0.996	-0.238	-0.419
	CO_02	3.427	[1; 5]	0.991	0.259	-0.646
	CO_03	3.655	[1; 5]	0.992	0.430	-0.829
Adoption intention	AD_01	4.023	[1; 5]	0.928	1.210	-1.046
	AD_02	3.776	[1; 5]	0.972	0.360	-0.712
	AD_03	3.845	[1; 5]	0.925	0.869	-0.785
Technology use	USE_01	3.983	[1; 7]	1.610	-0.894	-0.063

*Note:* Scores for perceived usefulness were updated for minor differences, as compared to Richter, Schubring, Hauff, Ringle and Sarstedt [1].

In addition, the data includes demographic information. The sample has an equal gender representation: 49% males (coded 1) and 51% females (coded 2). Age was measured in years. The participants were on average 41 years old (35% of respondents were below 30, 50% between 30 to 61, and 15% were 62 years old or older). Education was measured as the highest education achieved: 3% did not complete 8 years of schooling (coded 1), 6% had a secondary school education with completed apprenticeship (coded 2), 22% had a secondary school / junior high school / senior high school / technical school / commercial school education (coded 3), 26% had a qualification for university entrance (coded 4), 20% a bachelor's degree (coded 5), and 22% a master's degree (coded 6). To identify whether a respondent belonged to the target group of the study, they had to either own an e-book reader or use other devices to read electronic books or media. E-book reader ownership is provided in the dataset (and coded as: 1 = Yes; 2 = No).

## 3 Experimental Design, Materials and Methods

The following tables contain key results for evaluation purposes [16]. Table 3, Table 4, Table 5 to 6 include the results of a series of statistical analyses that verify convergent validity, discriminant validity and internal consistency reliability of the measurement models and the explanatory and predictive power of the structural model in accordance with guidelines provided by Hair, Hult, Ringle and Sarstedt [16]. The respective estimations were done with the statistical software package SmartPLS (version 4.0.8.8) [17]. Tables 7 and 8 contain the results of the NCA. The NCA included an assessment of the significance of the ceiling lines and the presentation of bottleneck tables, and was performed with both the statistical software *R* (using the NCA package developed by Jan Dul [5,6]) and SmartPLS 4^1^ [17].

**Table 3:** Convergent validity and internal consistency reliability.

Latent Variable	Item	Loadings	Indicator reliability	AVE	Composite reliability	Cronbach's α	ρ_A_
Emotional Value	EMV_01	0.891	0.794	0.853	0.946	0.914	0.917
	EMV_02	0.950	0.903				
	EMV_03	0.929	0.863				
Ease of Use	EOU_01	0.784	0.615	0.697	0.873	0.783	0.783
	EOU_02	0.878	0.771				
	EOU_03	0.840	0.706				
Perceived Usefulness	PU_01	0.722	0.521	0.642	0.842	0.723	0.753
	PU_02	0.819	0.671				
	PU_03	0.856	0.737				
Compatibility	CO_01	0.901	0.812	0.779	0.914	0.858	0.859
	CO_02	0.906	0.821				
	CO_03	0.840	0.706				
Adoption Intention	AD_01	0.933	0.870	0.889	0.960	0.938	0.939
	AD_02	0.935	0.874				
	AD_03	0.960	0.922				

*Source:* Adapted from Richter, Schubring, Hauff, Ringle and Sarstedt [1].

**Table 4:** Discriminant validity: HTMT criterion.

	Adoption intention	Compatibility	Ease of use	Emotional value	Technology use
Compatibility	0.631⁎				
Ease of use	0.524⁎	0.526⁎			
Emotional value	0.739⁎	0.675⁎	0.549⁎		
Technology use	0.642⁎	0.517⁎	0.375⁎	0.551⁎	
Perceived usefulness	0.637⁎	0.961^1^	0.594⁎	0.539⁎	0.489⁎

⁎ The 95% percentile-based confidence intervals do not include 0.9.

1 The discriminant validity evaluations point to a potential problem of discriminant validity between perceived usefulness and compatibility in this dataset, however, the discriminant validity between these two constructs has been conceptually discussed by authors when developing the scales [12], which is why we refrained from adaptations to the model. Researchers using this data in future applications are invited to explore this issue further; for this purpose, see also Franke and Sarstedt [18].

**Table 5:** Path coefficient estimates.

Paths	f^2^ effect sizes	Path coefficient	Indirect effects	Total effects
Emotional value ➔ Adoption intention	0.336	0.515^⁎⁎⁎^		
Emotional value ➔ Technology use	0.014	0.137	0.225^⁎⁎⁎^	0.362^⁎⁎⁎^
Ease of use ➔ Adoption intention	0.012	0.088		
Ease of use ➔ Technology use	0.000	0.01	0.038	0.049
Perceived usefulness ➔ Adoption intention	0.044	0.227*		
Perceived usefulness ➔ Technology use	0.002	0.05	0.099*	0.149
Compatibility ➔ Adoption intention	0.001	0.045		
Compatibility ➔ Technology use	0.006	0.107	0.020	0.127
Adoption intention ➔ Technology use	0.152	0.437^⁎⁎⁎^		

*p < 0.05; ^⁎⁎^p < 0.01; ^⁎⁎⁎^p < 0.001.

**Table 6:** Structural model explanatory and predictive power.

Construct	Item	R^2^	Q^2^_predict_	PLS-SEM - LM	
				RMSE	MAE
Adoption intention	AD_01	0.539	0.451	-0.020	-0.006
	AD_02		0.401	-0.048	-0.036
	AD_03		0.454	-0.039	-0.034
Technology use	USE_01	0.420	0.287	-0.044	-0.036

*Note:* PLS_predict_ results are based on three folds; the skewness and kurtosis values of the prediction errors are between -2 and +2.

**Table 7:** NCA effect sizes.

Construct	Adoption intention	Technology use
	CE-FDH	CE-FDH
Emotional value	0.214^⁎⁎⁎^	0.331^⁎⁎⁎^
Ease of use	0.151^⁎⁎^	0.235*
Perceived usefulness	0.119^⁎⁎^	0.243^⁎⁎^
Compatibility	0.082*	0.211^⁎⁎⁎^
Adoption intention		0.294^⁎⁎⁎^

*Note:* *p < 0.05; **p < 0.01; ***p < 0.001.

**Table 8:** Bottleneck table (percentages).

	Emotional value	Ease of use	Perceived usefulness	Compatibility	Adoption intention

*Bottleneck adoption intention*					
0	NN	NN	NN	NN	
10	NN	10.2	9.3	NN	
20	NN	10.2	9.3	NN	
30	NN	10.2	9.3	NN	
40	NN	10.2	9.3	NN	
50	NN	20.0	9.3	NN	
60	41.1	20.0	15.7	NN	
70	41.5	20.0	15.7	NN	
80	49.7	20.0	15.7	32.9	
90	49.7	20.0	15.7	32.9	
100	49.7	20.0	15.7	32.9	
*Bottleneck technology use*					
0	NN	NN	NN	NN	NN
10	NN	10.2	NN	NN	NN
20	NN	10.2	NN	NN	NN
30	NN	10.2	NN	NN	NN
40	49.7	20.0	15.7	25.5	33.8
50	49.7	20.0	15.7	25.5	33.8
60	49.7	20.0	15.7	33.7	33.8
70	49.7	20.5	48.1	33.7	33.8
80	49.7	20.5	48.1	33.7	33.8
90	49.7	60.2	66.2	33.7	75.0
100	49.7	60.2	66.2	33.7	75.0

*Note:* Percentage values in the bottleneck tables have minor deviations from those reported in Richter, Schubring, Hauff, Ringle and Sarstedt [1]. This is due to the former being based on an extraction of latent variable scores rounded to the third decimal place from SmartPLS 3 and the following import to the software R. Replicating these results with SmartPLS 4 with more decimal places gives the above results.

The data was collected with an online survey based on a questionnaire developed for the purpose of investigating key determinants of consumers’ technology adoption intention and use with an extended TAM. More specifically, it provides information on reasons why French consumers decide to adopt and use e-book readers, therewith being technology adopters. Technology adopters are broadly defined as an aggregate of current adopters, those consumers who have already embraced a given technology, and prospective adopters, those consumers who are likely to embrace said technology soon [19].

Existing well-established measurement models from academic research within the field of technology acceptance and innovation diffusion were adapted and used to measure each construct. Furthermore, best practices and guidelines for designing and conducting market research as prescribed by Sarstedt and Mooi [20] were adhered to. Perceived usefulness, compatibility, emotional value, ease of use, and adoption intention were each measured with three items and operationalized reflectively. Technology use was measured with a single item (USE_1). Table 1 provides – in addition to the operationalizations – the sources of the constructs included in the data.

The online survey was programmed in the web-based application software LimeSurvey.^2^ Prior to proceeding with the data collection, two pre-tests were performed: the questionnaire was reviewed by members of the Institute of Human Resource Management and Organizations at the Hamburg University of Technology; a link to the online survey was distributed to 45 potential respondents and resulted in 33 completed questionnaires. Based on feedback from both sources, adjustments were made to improve the quality and clarity of the questionnaire.

Survey distribution in France began on the 4^th^ of March 2014 and ended on 12^th^ of March 2014. This was done in collaboration with the international market research agency Harris Interactive^3^ and was based on a quota sampling technique with pre-defined criteria that aimed to ensure an adequate and sufficient representation of different sociodemographic strata among the French population spread across the entirety of France. Following from the involvement of a professional market research data provider, there was a very low drop-out rate of 4%. Another 4% were removed from the datafile due to suspicious response patterns.

## Ethics Statements

Survey participants have given an informed consent prior to completing the survey and their responses were recorded completely anonymously. No personal data that could make a participant directly identifiable has been gathered and/or retained. The online survey software application Limesurvey is used to develop anonymous surveys and does not share data with third parties. The market research company Harris Interactive AG that facilitated the data collection process implements a privacy policy in compliance with GDPR. Ethical approval was not necessary for this study.

## CRediT authorship contribution statement

**Nicole Franziska Richter:** Conceptualization, Writing – review & editing, Formal analysis, Validation, Supervision. **Sven Hauff:** Writing – review & editing, Formal analysis, Validation. **Aleksandar Evgeniev Kolev:** Writing – original draft, Formal analysis, Validation, Visualization. **Sandra Schubring:** Conceptualization, Investigation, Methodology, Resources.

## Declaration of Competing Interest

The authors declare that they have no known competing financial interests or personal relationships that could have appeared to influence the work reported in this paper.

## Data Availability

Extended TAM (Original data) (Mendeley Data).

## Footnotes

1 SmartPLS 4 includes selected NCA capabilities that are being updated on a regular basis; users of SmartPLS will also find the dataset as an example in the software's environment.

2 For more information visit: http://www.limesurvey.org/de/.

3 For more information visit: http://www.harris-interactive.de/.
